# Supplementary material for: Direct Current Electrical Stimulation Shifts THP-1-Derived Macrophage Polarization towards Pro-Regenerative M2 Phenotype
Source: Int J Mol Sci. 2024 Jul 2;25(13):7272. doi: 10.3390/ijms25137272 (PMC11242703; doi:10.3390/ijms25137272)
Supplement: Supplementary file 1 [file ijms-25-07272-s001.zip › ijms-3046230-supplementary.pdf]

SUPPLEMENTARY MATERIALS

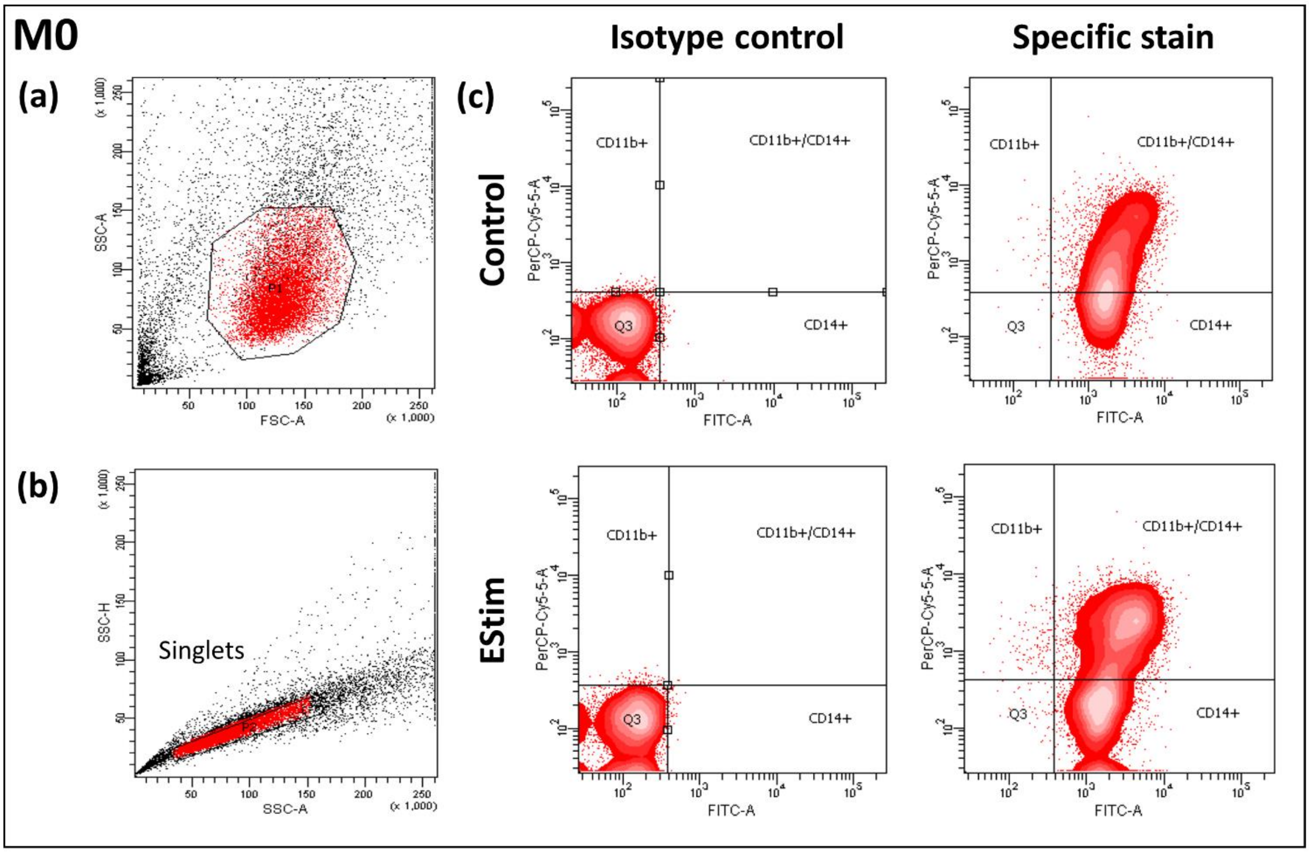

Supplementary Figure S1. Assessment of cell surface markers of THP-1-derived macrophages by flow cytometry. SSC and FSC gating (a) and doublet exclusion (b) dot plots. Isotype control staining and specific antibody staining contour maps (c) from M0 macrophages exposed (or not, control group) for 3 days to DC EStim. SSC, side scatter; FSC, forward scatter; DC EStim, direct current electrical stimulation. The mixture of fluorochrome-conjugated antibodies consisted of CD11b PE-Cy 5, CD14 FITC, CD80 BV421, CD86 APC, and CD206 PE. A mixture of their corresponding fluorochrome-conjugated isotypes was employed as negative control.

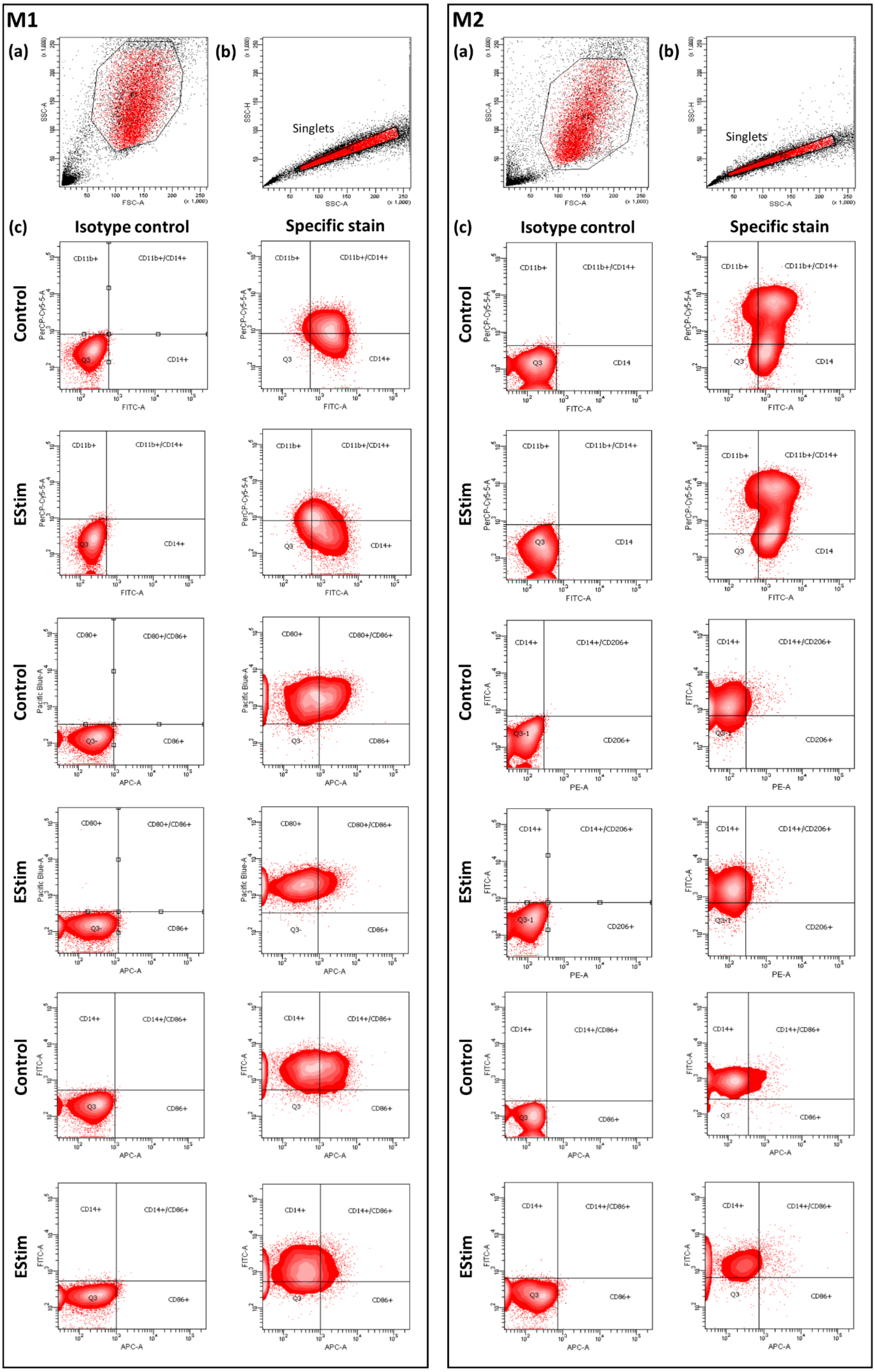
M1
M2
(a)
(b)
(c)
Singlets
Isotype control
Specific stain
Control
EStim
CD11b+
CD11b+/CD14+
CD14+
CD14
CD80+
CD80+/CD86+
CD86+
CD14+/CD206+
CD206+
CD14+/CD86+
Q3
FSC-A
SSC-A
SSC-H
FITC-A
APC-A
PE-A
PerCP-Cy5-5-A
Pacific Blue-A

Supplementary Figure S2. Assessment of cell surface markers of THP-1-derived macrophages by flow cytometry. SSC and FSC gating (a) and doublet exclusion (b) dot plots. Isotype control staining and specific antibody staining contour maps (c) from M1 and M2 macrophages exposed (or not, control group) for 3 days to DC EStim. SSC, side scatter; FSC, forward scatter; DC EStim, direct current electrical stimulation. The mixture of fluorochrome-conjugated antibodies consisted of CD11b PE-Cy 5, CD14 FITC, CD80 BV421, CD86 APC, and CD206 PE. A mixture of their corresponding fluorochrome-conjugated isotypes was employed as negative control.

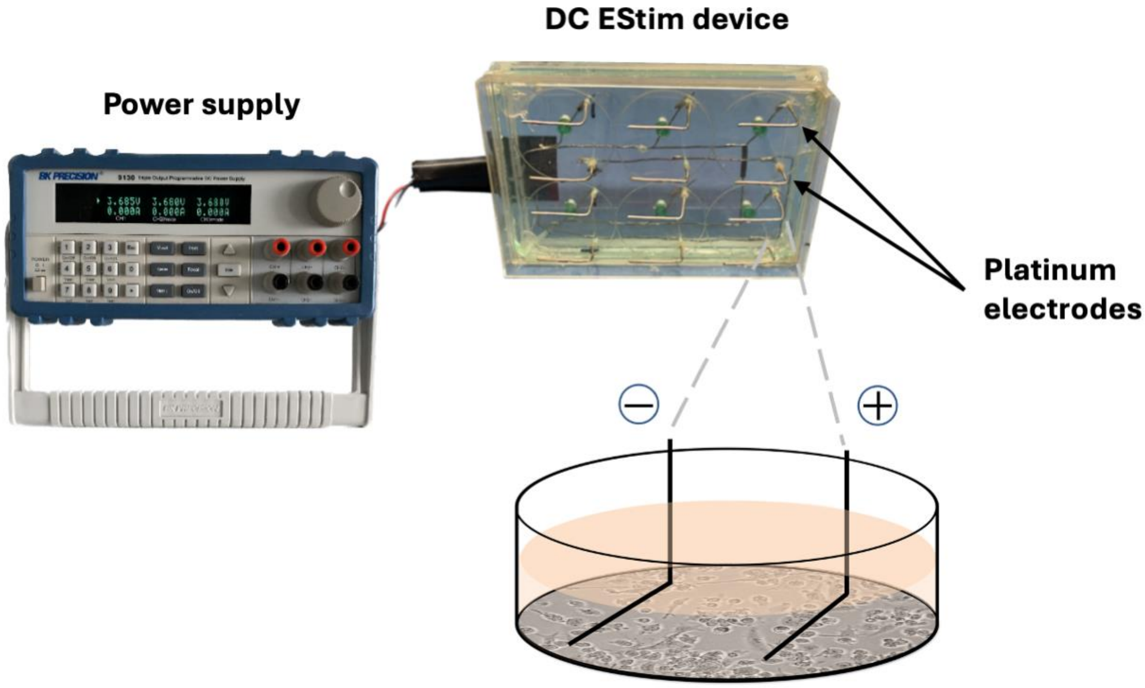

Supplementary Figure S3. Electrical stimulation setup. During the polarization stage (three days after priming and resting), M0, M1, and M2 macrophages were treated with 100 mV/mm direct current electrical stimulation (DC EStim) for 1 h per day using a custom-made DC EStim device.
